# Associations between daily composition of 24 h physical behavior with affective states and working memory

**DOI:** 10.1038/s41598-025-99266-4

**Published:** 2025-04-25

**Authors:** Flora Le, Viola Mattern, Peter J. Johansson, Pasan Hettiarachchi, Ulrich Ebner-Priemer, Joshua F. Wiley, Dorothea Dumuid, Marco Giurgiu

**Affiliations:** 1 School of Psychological Sciences and Turner Institute for Brain and Mental Health, Monash University, Victoria, Australia; 2 Institute of Sports and Sports Science, Karlsruhe Institute of Technology (KIT), Engler-Bunte-Ring 15, 76131 Karlsruhe, Germany; 3 Occupational and Environmental Medicine, Uppsala University Hospital, Uppsala, Sweden; 4 Occupational and Environmental Medicine, Department of Medical Sciences, Uppsala University, Uppsala, Sweden; 5 Alliance for Research in Exercise, Nutrition and Activity, Allied Health & Human Performance, University of South Australia, Adelaide, Australia

**Keywords:** Physical activity, Sedentary behavior, Standing, Sleep, Affect, Working memory, Bayesian multilevel compositional data analysis, Risk factors, Human behaviour

## Abstract

The daily association between 24-hour physical behavior compositions (moderate-to-vigorous physical activity (MVPA), light physical activity (LPA), standing, sedentary, and sleep) and psychological outcomes—such as momentary affective state assessments and working memory—remains understudied. We investigated whether the daily 24-hour compositions, particularly MVPA and SB considering the remaining behaviors, are associated with affective states and working memory. We conducted an ambulatory assessment study with 199 university employees. Physical behaviors were measured continuously via thigh-worn accelerometers throughout the day. Affective states (i.e., valence, energetic arousal, and calmness) and working memory performance (i.e., numeric updating task) were captured up to six times a day via electronic diaries and tasks on a smartphone. We conducted Bayesian multilevel compositional data analysis to analyze within-person, and between-person associations of 24-hour physical behavior composition with affective states, and working memory. Aggregated same-day outcomes were used for main analyses to capture concurrent associations, and next-day outcomes were used for exploratory analyses to capture prospective associations. Concurrent analyses showed that higher moderate-to-vigorous physical activity relative to the remaining physical behaviors was associated with 2.49 [95%CI 1.00, 4.06] higher valence and 3.65 [95%CI 2.11, 5.28] higher energetic arousal (but not calmness) ratings at the within-person, but not at the between-person level. Sedentary behavior relative to the remaining physical behaviors was not associated with any affective states. Spending more time in moderate-to-vigorous physical activity, followed by light physical activity, and standing, each at the expense of the other behaviors was associated with higher affective state ratings on the same day (*between-person*: ≥1.29 [0.19, 2.51] higher valence, 1.23 [0.04, 2.40] higher calmness; *within-person*: ≥0.62 [0.04, 1.22] higher valence, ≥ 1.10 [0.63, 1.58] higher energetic arousal, ≥ 0.95 [0.18, 1.74] higher calmness). The 24-hour physical behavior composition was not associated with working memory. Findings underline the importance of the 24-hour composition of physical behavior for mental health, by demonstrating significant concurrent associations with affective states. Even small reallocations of behaviors may positively influence affective states, providing valuable insights for the development of future interventions.

**Supplementary Information:**

The online version contains supplementary material available at 10.1038/s41598-025-99266-4.

## Introduction

Physical inactivity is a leading risk factor for all-cause mortality, chronic diseases, depression, and dementia, whereas being physically active can influence both affective and cognitive parameters positively^1–3^. From a theoretical perspective, physical behavior can have consequences on affective states (e.g., valence or energetic aorusal) and cognitive outcomes (e.g., executive functions such as working memory) but can also be determined in a dual-process manner^4,5^. Dual-process models assume that behavior and decision-making are influenced by both an automatic, affect-based system and cognitively-controlled system^5,6^. In this context, the cognitive-controlled system can be assessed by working memory performance, which is characterized as one of the three central core executive functions and defined as holding information in mind and mentally working with it^6^. To overcome physical inactivity and promote health, the latest physical activity (PA) guidelines for adults by the World Health Organization recommend a minimum of 150 min (up to 300 min) of moderate intensity physical activity or 75 min (up to 150 min) of vigorous-intensity physical activity^3^. However, the prevalence of physical inactivity increased from 23.4% in 2000 to 31.3% in 2022^7^, emphasizes the need to reduce sedentary time and towards more active lifestyle. Sedentary behavior (SB) (i.e., any sitting, reclining, or lying waking behavior with an energy expenditure of no more than 1.5 metabolic equivalents^8^) might be a risk factor for several health conditions^9,10^. Notably, the negative health effects of SB are dependent on the current physical activity level^11^. These findings support that a balance of time spent in different physical behaviors throughout the full 24-hour (24-h) day is essential.

In line with this approach, the 24-hour perspective has become a central part of conceptual models and approaches. In particular, Pedišić^12^introduced first the Activity Balance Model, a theoretical framework for investigating associations of 24-hour physical behavior with health outcomes, and a couple of years later together with his team the Framework for Viable Integrative Research in Time-Use Epidemiology^13^. A couple of years later, Rosenberger et al. (2019) introduced the 24-h Activity Cycle that focuses on the interrelationship between different physical behaviors such as sleep period, SB, standing, light PA (LPA), and moderate-to-vigorous PA (MVPA). The 24-h Activity Cycle is dynamic – these physical behaviors are not independent of each other, because increased time in one behavior must result in decreased time spent elsewhere^14^. These behaviors should therefore be analyzed relative to each other, as an integrated composition, rather than as individual behaviors^15,16^.

Physical behaviors are key modifiable lifestyle factors for the prevention and management of mental disorders^17^, and cognitive health. While studies remain inconclusive about the associations between these behaviors and cognitive health^18^, empirical evidence suggests that being physically active and reducing sedentary time are associated with lower risk of depression^19–21^. Specifically, the reallocation of time to MVPA from other behaviors has been associated with better mental health outcomes^19,22,23^. Mental health is an umbrella term covering several constructs, such as well-being, emotions, affective states, mood, or cognitive indicators. Affective states, in particular, are central indicators of mental well-being in healthy populations^24^and are altered in mental disorders, such as major depression^25^. According to the literature, there is an ongoing discussion about the operationalization of affective states (i.e., two-dimensional structure^26^vs. three-dimensional structure^27^). Based on the analyses reported by Wilhelm and Schoebi^28^, a three-dimensional approach (including valence, energetic arousal, and calmness) is highly sensitive to capturing within-person changes in affective states. Despite the established associations between independent physical behaviors and affective experience^29–31^, the compositional associations between physical behaviors in the 24-h Activity Cycle and affect remain not well understood.

To our knowledge, only two studies examined the association between 24-h physical behaviors and affect. One analysis found that more MVPA relative to LPA or SB was associated with higher high arousal positive affect^32^. This analysis was, however, cross-sectional, thus not capturing the within-person changes of physical behaviors and affect throughout the day. Most studies on the compositional effects of physical behaviors on mental health outcomes are cross-sectional, which is a methodological shortcoming since mental health outcomes such as affective states undergo a diurnal variation^33^. Ambulatory assessments which are sensitive enough to capture fluctuations are required to gather insights into the natural variation (e.g., across days) of affective state^34^. Ambulatory assessments can be combined with continuous time-stamped behavior measurement via wearables over several continuous days, enabling the examination of both between-person associations (i.e., differences between individuals) and within-person associations between affective states and physical behaviors in real-time during participants’ everyday lives. Insights into how daily, between- and within-person reallocations of time behaviors are associated with affect could improve guidelines promoting physical behaviors for affective and mental health improvements. The only study that focused on within-person associations between affect valence and waketime movement behavior by using AA was conducted by Bourke et al.^35^. The authors revealed a weak within-person association between movement behavior compositions and affective valence, while showing that less sedentary time than usual and instead of engaging in PA was significantly related to more positive affective valence^35^.

In this study, we examine the daily associations between 24-h physical behavior compositions and affective states (i.e., valence, calmness, energetic arousal) as well as working memory performance. Extending previous studies, we consider a five-part composition of MVPA, LPA, standing, SB, and sleep period, with a focus on MVPA and SB, considering their trade-offs with other behaviours. Based on previous studies^32,36^, we hypothesize that more MVPA relative to the remaining behaviours (SB, LPA, and sleep period) is positively associated with energetic arousal (1a), valence (1b), working memory (1c), and negatively associated with calmness (1 d) on both within-person and between-person levels. Furthermore, we hypothesize that more SB relative to the remaining behaviours (LPA, MVPA, and sleep period) is negatively associated with energetic arousal (2a), valence (2b), working memory (2c), and calmness (2 d), on both within-person and between-person levels. In exploratory analyses, we examine how physical behavior compositions are prospectively associated with affective states and working memory performance of the subsequent day to explore compositional effects that last over time. Finally, we conducted a series of exploratory reallocation models across different time frames (i.e., 1 to 60 min).

## Methods

### Participants

Between October 2022 and December 2023, we recruited 222 university employees, a population shown to be high risk for SB^37^. The job characteristics in our sample included scientific staff, technical employees, and administrative secretaries, all recruited participants reported that they spent most of their time in a sedentary position. Moreover, participants were included only if they were able to perform daily life activities, indicating no physical injuries or mental health issues at the time of the study. We excluded 23 participants from the analyses: *N* = 19 did not meet the accelerometer wear time criteria, *N* = 3 discontinued the study, and *N* = 1 experienced technical issues. Consequently, our final sample comprised 199 participants (55.3% female), with an average age of 35.8 ± 10.7 years and an average body mass index (BMI) of 24 ± 3.3 kg/m² (details in Table 1). The Ethics Committee of the Karlsruhe Institute of Technology (KIT) approved the study. All study procedures were performed in accordance with the Declaration of Helsinki. All participants received detailed written and verbal information about the study procedures before giving their written informed consent. They were free to withdraw from the study at any time. As an incentive, participants were given a fitness tracker valued at €100.

**Table 1:** Participants’ characteristics (*N* = 199; female 55.3%).

Variable	Mean ± SD^1^	Minimum	Maximum
Age [yrs.]	35.84 ± 10.7	20.18	64.74
BMI [kg/m^2^]	23.97 ± 3.3	17.87	35.25
VO2_Max_ [l/min] ^a^	2.97 ± 0.7	1.47	5.36
Valence [0–100] ^b^	65.69 ± 12.2	39.68	98.45
Energetic Arousal [0–100] ^b^	56.25 ± 12.0	25.89	89.09
Calmness [0–100] ^b^	60.87 ± 12.8	33.19	96.37
Working Memory [0–100%] ^c^	88.55 ± 9.0	48.61	99.69
Wear Time Accelerometer [h/day] ^c^	20.4 ± 2.7	10.63	23.74
Sleep period [h/day] ^d^	7.95 ± 1.1	4.27	12.70
Sedentary time [h/day] ^d^	10.5 ± 1.1	6.68	14.14
Standing [h/day] ^d^	2.79 ± 0.84	1.31	5.87
LPA [h/day] ^d^	1.15 ± 0.31	0.33	2.13
MVPA [h/day] ^d^	1.35 ± 0.42	0.51	2.89

^1^ standard deviation;.

^a^ data available from *N* = 196 participants.

^b^ assessed via e-diary, aggregated per participant per day (data available form *N* = 194 participants);.

^c^ assessed via mobile task, aggregated per participant per day (data available form *N* = 191 participants);.

^d^ aggregated per participant and day.

## Study procedures

The present study is a secondary analyses of a within-person encouragement design (see for details^38^). After recruiting via flyers, mailing, and word of mouth, participants completed an initial in-person session of approximately three hours. During this session, participants received written and verbal information regarding the study procedures, were instructed and trained on the technical equipment, completed cognitive tasks, filled in questionnaires, and performed an incremental treadmill test to volitional exhaustion with a 6-2−1protocol (i.e., starting with 6 km/h and changing the speed by 1 km/h after every two minutes). Following the in-person session, participants started with the ambulatory assessment phase for 15 working days. If the participants were not able to participate continuously for 15 working days (e.g., due to sickness or vacation), the study period was extended until data from at least 15 working days were collected. Participants were equipped with a study smartphone (Nokia G50 or 6, Nokia Corporation, Espoo, Finland, nokia.com) and were instructed to wear the move 4 accelerometer (movisens GmbH, Karlsruhe, Germany, movisens.com) on their right thigh continuously for 24-h per day (Monday to Friday). The weekend days were designated as break days without assessments to maintain motivation and ensure compliance. The study was implemented by using a within-person encouragement design^39^. In particular, each working day between 8:40 am and 9:40 pm, the smartphone prompted participants up to six times per day to rate their momentary affective states and to perform the working memory task. Notably, half of the prompts were provided following a sedentary bout (i.e., 30 min uninterrupted sitting/lying). We implemented a sedentary-triggered algorithm^40^, i.e., prompting e-diary ratings after 30 consecutive minutes in a sitting/lying body position. Participants could delay the prompts up to 15 min, with a maximum of three delays of five minutes each. To reduce the participants burden, 30-minute time-outs were implemented, which means after a completed e-diary rating, the participants were not prompted for at least 30 min.

## Assessment of 24-hour physical behavior

The Move 4 accelerometer (movisens GmbH, Karlsruhe, Germany, movisens.com) captured physical behaviors with a range of ± 16 g and a sampling frequency of 64 Hz. Raw acceleration data were stored on an internal memory card and pre-processed by using ActiPASS 2024.05 software^41^. Previous studies validated the software across different thigh-worn accelerometer brands and indicated excellent accuracy for both wake-time physical behaviors (> 90%) and sleep (84%)^42–45^. ActiPASS implements algorithms for non-wear, sleep detection (i.e., a two-stage sleep detection process), posture, and activity intensity derived from cadence measures^46,47^. We calculated the following five compositions as average minutes/day: Sleep period, SB (sitting or lying episodes outside of sleep intervals), standing, LPA (ambulatory movement without purposeful walking, walking with cadence < 100 steps/min), and MVPA (running, cycling, inclined stepping, walking with cadence ≥ 100 steps/min). We included only participants and days that fulfilled the following accelerometer criteria: i) participants with at least three valid wear time days (≥ 20 h of wear/day), ii) ≥ 1 period of walking detection, iii) and days with > 0 min of sleep.

## Assessment of affective states

To assess momentary affective states over time, we used a short scale consisting of six items developed and validated by Wilhelm and Schoebi^28^. The scale captures three basic affective state dimensions, including valence (V), energetic arousal (EA), and calmness (C). Psychometric properties in our sample with within-subject reliability coefficients ranging between 0.58 and 0.91. The six bipolar items were presented to participants in a reversed polarity and mixed order in German translation on visual analog scales (0–100). The items include: EA1 – tired to awake; V1 – content to discontent; C1 – agitated to calm; EA2 – full energy to without energy; V2 – unwell to well; C2 – relaxed to tense. The questions were presented on the smartphone via the application movisensXS (version 1.5.24; movisens.com). Affect was averaged across the day to obtain a daily average affect value.

## Assessment of working memory performance

**Fig. 1:**
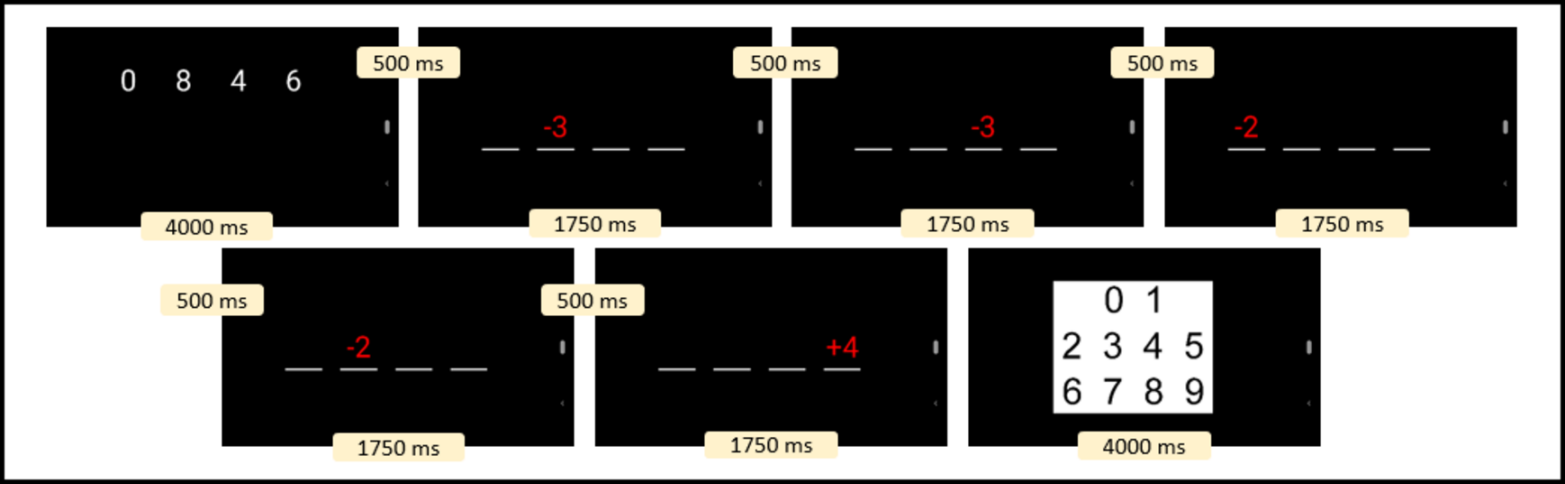
Example of the numerical updating task.

Participants completed a numerical updating task^48^. The task involved memorizing a horizontal array of four digits and then updating these digits based on arithmetic operations (i.e., addition and subtraction in the range of +/- 1 to 8) presented in a random order. The four starting digits were displayed for 4000 ms, followed by an inter-stimulus interval of 500 ms. In total, participants completed nine trials at each measurement occasion. During each trial, five updating operations had to be applied (i.e., each of the four digits had to be processed at least once and one digit twice) with a presentation time of 1750 ms. The presentation time for arithmetic operations was reduced by 250 ms after the 3rd and 6 th trials. Participants had 4000 ms time per digit to enter the result via tapping on a digit block (see Fig. 1). At each measurement occasion, the percentage of correct responses was computed. The within-subject reliability coefficient in our sample was ω = 0.57. The entire task took approximately three and a half minutes to complete. The task was presented on the smartphone via the software Presentation (version 3.0.4; Neurobehavioral Systems, Inc., https://www.neurobs.com/).

### Statistical analyses

Bayesian multilevel compositional data analysis^49^ was used to account for the relative and constrained nature of 24-h physical behaviors by quantifying the expected change in outcome when reallocating time to a specific behavior from one or more other behaviors. Isometric-logratio (*ilr*) coordinates were employed to account for the perfect multicollinearity of direct composition measure (as time spent in behaviors always sums to 24-h) and account for their interdependence. A specific sequential binary partition was used to create the *ilr*pivot coordinates^50,51^. This partitioning produced two sets of *ilr* pivot coordinates with the two first coordinates representing the MVPA and SB, respectively, relative to the remaining behaviors. This was done at both between- and within-person levels, with raw compositional data decomposed into the between- and within-person levels before calculating the ilr coordinates. The between-person *ilr* pivot coordinates represent the average time spent in the 24-h composition, whereas the within-person *ilr* pivot coordinates are the mean-centered deviates from an individual’s average 24-h composition on a given day. We defined a 24-h day from midnight to midnight.

Bayesian compositional multilevel models (Le et al., 2024) were fitted, with each model including average ratings of affect states and performance of working memory as respective outcomes and between-person and within-person physical behavior composition (expressed as *ilr* coordinates) as predictors. Same-day outcomes (i.e., aggregated values for each physical behavior on a day level) were used for main analyses to capture concurrent associations, and next-day outcomes were used for exploratory analyses to capture prospective associations. A random intercept and random slopes of the within- person compositions (expressed as *ilr* coordinates) by participants were included. All models adjusted for daily factors, including previous-day lag outcome and total wear time, alongside baseline age, sex, and body mass index. Models were fitted with weakly-informative priors, 4 chains, each with 2000 iterations including 1000 warmups (total of 4000 post-warmup draws). Model convergence was evaluated and confirmed using diagnostic statistic $\:\widehat{R}$< 1.05 and effective sample size > 400^52^. Coefficients of the *ilr* coordinates representing associations of MVPA and sedentary, respectively, relative to the remaining behaviors, with the respective outcomes were reported.

Bayesian compositional multilevel substitution analysis^49^then estimated the difference in affective states and working memory for all possible pairwise time reallocations between behaviors for one-minute increments from 1 to 60 min. All estimates are relative to the mean composition among included participants. Due to the non-linear nature of the reverse transformation from isometric log-ratios to minutes/day, the reallocations were repeated both for adding to, and deducting from, time spent in each behavior. Unstandardized posterior mean differences were computed, and statistical significance of individual parameters was set as Bayesian 95% posterior credible intervals (CIs) not including zero. Complete data were used for all analyses because methods for imputing multilevel compositional data are not yet well developed. A sensitivity analysis was also conducted in participants with at least 3 ratings of affect per day, which showed similar results to the main sample. We included the full sensitivity analysis results in the supplementary results. Analyses were performed in R (R Core Team, 2023) and Stan (Stan Development Team, 2024) (via cmdstanr), using package multilevelcoda^49,53^for data pre-processing, model estimation, post-hoc analyses, and brms^54^ for back-end model estimation. Analysis code and supplementary results are available at https://github.com/florale/emia-24 h-affect-wm.

## Results

### Sample characteristics

In total, we analyzed 2,352 days of 199 participants (i.e., on average 11.81 days/participant, ranging from 3 to 22 days). Sample characteristics are in Table 1. 55.3% (*n* = 110) of the sample were female with a mean age of 35.84 years ± 10.7 (range: 20–65), mean BMI of 23,97 kg/m³ ± 3.3 (range 17.87–35.25), and an average VO2 max of 2.97 l/min. Mean daily wear time across the study period was 20.4 h ± 2.7. The average 24-h composition, normalized to a 24-h day, was 7.95 h in sleep period, 10.5 h sedentary, 2.79 h standing, 1.15 LPA, and 1.25 h MVPA. Across the included 2,352 days, in 13,329 situations participants were prompted via smartphone to answer momentary affective states and to perform the mobile working memory task. In total, 9,440 prompts were answered (i.e., a compliance rate of 69.88% on a person level). On average participants answered 4.4 ratings per day. In 82.4% of all days, participants answered at least three ratings. The full sample had an average valence [scale 0–100] of 65.69 (range: 39.68–98.45), energetic arousal of 56.25 (range: 25.89–89.09), calmness of 60.87 (range: 33.19–96.37), and working memory [scale 0–100%] of 88.55 (range 48.61–99.69; and thus, indicating ceiling effects). The available sample for the outcomes ranged from *n* = 191 (working memory) to *n* = 196 (valence, energetic arousal, calmness).

### Concurrent associations between 24-h physical behavior compositions and affective States

Supporting hypothesis 1a and 1b, a higher ratio of MVPA relative to the remaining behaviors predicted higher valence (2.49 [95% CI 1.00, 4.06]) and higher energetic arousal (3.65 [2.11, 5.28]) at the within-person level. However, no associations were identified on the between-person level. Moreover, MVPA relative to the remaining behaviors was not associated with calmness. In contrast to our hypothesis 2, SB relative to the remaining behaviors was not associated with any type of affective states (see Table 2).

**Table 2:** Compositional associations between daily physical behaviors, affective States, and working memory.

	Valence	Energetic Arousal	Calmness	Working Memory
**Concurrent associations**				
***Between-person level***				
Pivot coordinate of MVPA vs. remaining behaviors	−2.27 [−8.20, 3.89]	−3.24 [−9.44, 2.77]	−3.93 [−10.01, 2.32]	−3.51 [−7.44, 0.25]
Pivot coordinate of Sedentary vs. remaining behaviors	−3.20 [−15.08, 9.02]	−6.28 [−17.80, 4.89]	2.58 [−9.53, 14.22]	−0.32 [−7.69, 7.51]
***Within-person level***				
*Within-person* pivot coordinate of MVPA vs. remaining behaviors	**2.49** **[1.00**,** 4.06]**	**3.65** **[2.11**,** 5.28]**	−0.14 [−1.80, 1.46]	0.19 [−0.59, 0.95]
*Within-person* pivot coordinate of Sedentary vs. remaining behaviors	−1.35 [−3.45, 0.60]	−1.17 [−3.63, 1.24]	−1.27 [−3.50, 0.88]	−0.62 [−1.72, 0.50]
**Prospective associations**				
***Between-person level***				
Pivot coordinate of MVPA vs. remaining behaviors	−2.05 [−8.27, 4.22]	−3.48 [−9.34, 2.58]	−3.53 [−9.84, 3.06]	−1.18 [−5.23, 3.04]
Pivot coordinate of Sedentary vs. remaining behaviors	−2.42 [−13.80, 9.16]	−4.42 [−15.08, 5.95]	2.01 [−9.47, 13.41]	−0.37 [−8.16, 7.25]
***Within-person level***				
Pivot coordinate of MVPA vs. remaining behaviors	0.23 [−1.07, 1.54]	0.06 [−1.62, 1.68]	−1.24 [−2.72, 0.25]	−0.08 [−0.95, 0.86]
Pivot coordinate of Sedentary vs. remaining behaviors	0.33 [−1.55, 2.23]	−0.60 [−2.74, 1.50]	−1.01 [−3.03, 1.09]	−0.57 [−1.86, 0.66]

*Notes*. MVPA = moderate-to-vigorous physical activity. Values are unstandardised mean [unstandardised 95% credible intervals]. Bold values indicate 95% credible intervals not containing 0. Covariates included age, sex, body mass index, daily total wear time, and previous-day lag outcome.

## Concurrent reallocation between 24-h physical behavior compositions and affective States

### Valence

**Fig. 2:**
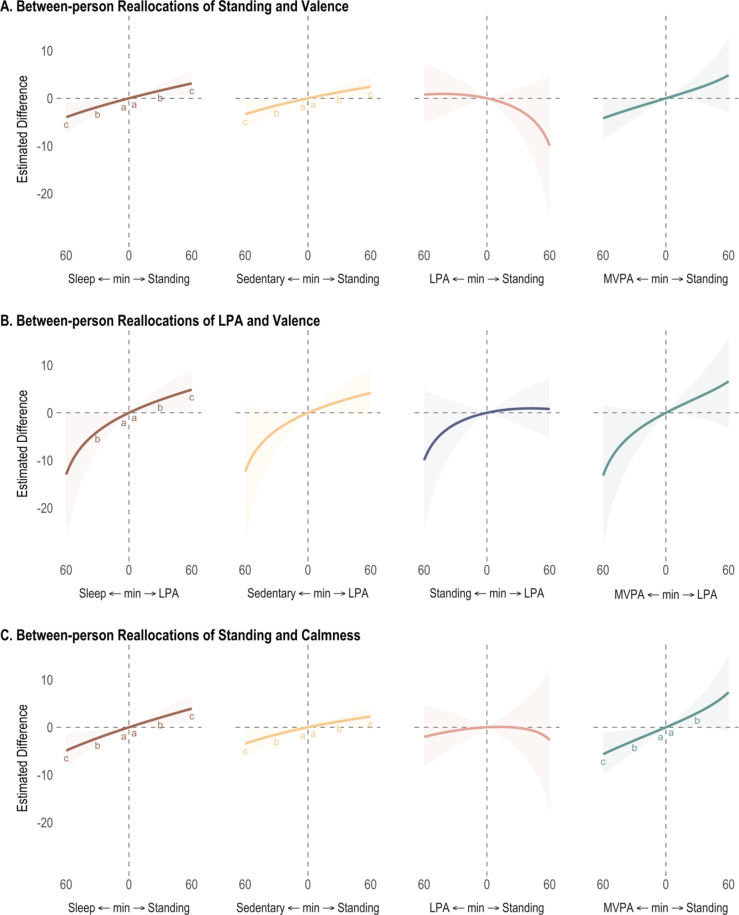
Estimated concurrent differences in affect at the between-person level for 24-h physical behavior reallocations from 1 to 60 min. Sleep = sleep period, MVPA = moderate-to-vigorous physical activity, LPA = light physical activity. Symbols indicate 95% credible intervals not containing 0 for ^a^5 minutes, ^b^30 minutes, and ^c^60 minutes. Covariates included age, sex, body mass index, daily total wear time, and previous-day lag outcome.

**Fig. 3:**
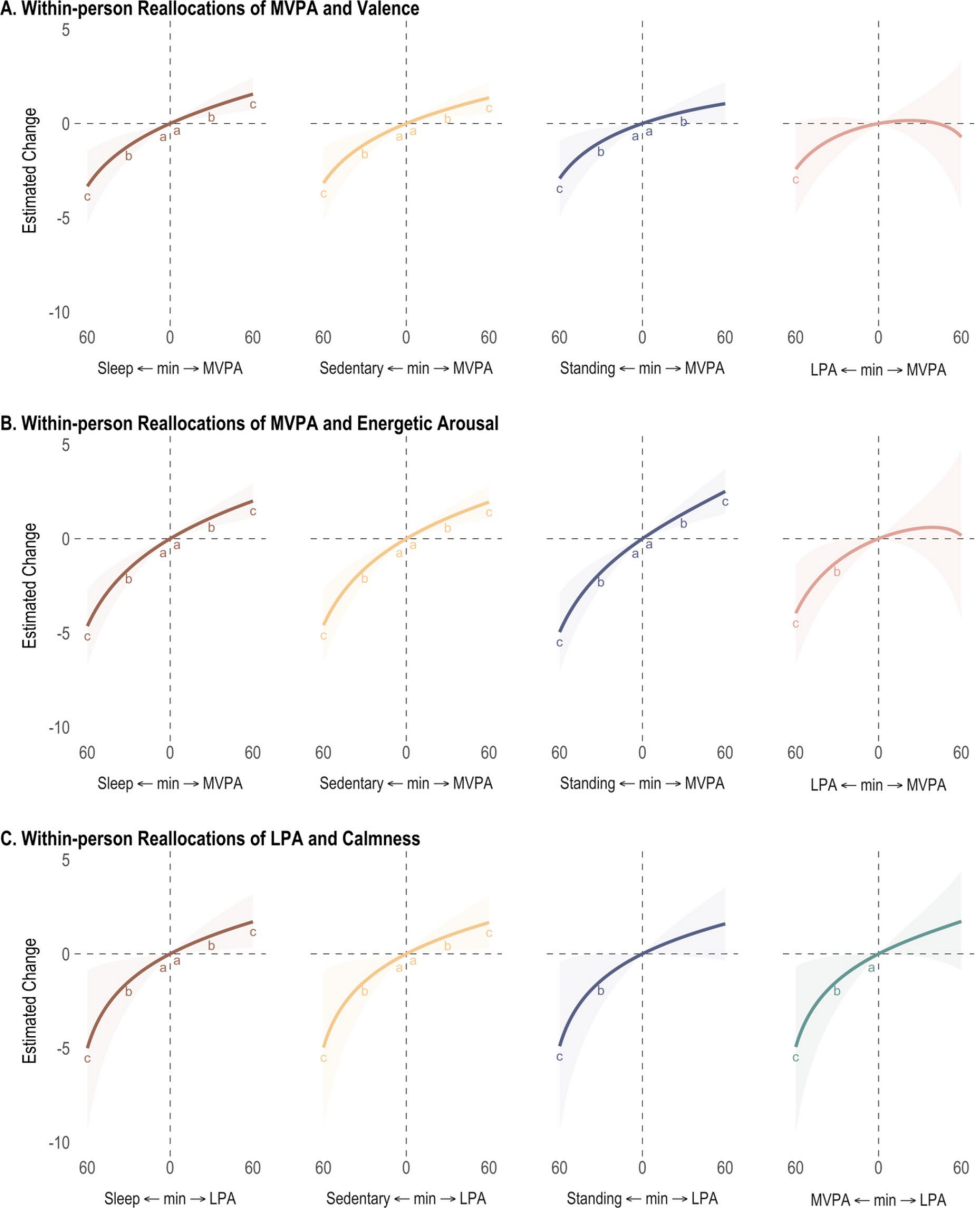
Estimated concurrent differences in affect at the within-person level for 24-h physical behavior reallocations from 1 to 60 min. Sleep = sleep period, MVPA = moderate-to-vigorous physical activity, LPA = light physical activity. Letters indicate 95% credible intervals not containing 0 for ^a^5 minutes, ^b^30 minutes, and ^c^60 minutes. Covariates included age, sex, body mass index, daily total wear time, and previous-day lag outcome.

 Reallocations of 24-h behaviors were concurrently associated with valence at both *between-person* and *within-person* levels (Table 3; Fig. 2 Panel A and B, and Fig. 3 Panel A). At the between-person level, individuals who spent + 30-minute more standing at the expense sleep period or SB experienced higher valence (+ 1.63 [95% CI 0.33, 2.96] and + 1.29 [95% CI 0.19, 2.51], respectively). Additionally, those who spent + 30-minute more LPA at the expense of sleep period experienced 2.71 [0.17, 5.19] higher valence. The opposite reallocations (less LPA compensated by sleep or SB) were also associated with lower valence, with estimates ranging from − 1.51 [−2.94, −0.21] to −3.94 [−7.80, −0.03]. At the within-person level, reallocations involving MVPA were most associated with valence. More time in MVPA at the expense of other behaviors (sleep period, sedentary, and standing) was associated with higher valence. For 30 min more MVPA, estimates ranged from + 0.62 [0.04, 1.2] to + 0.86 [0.39, 1.34], whereas for 30 min less MVPA, estimates ranged from − 0.97 [−1.73, −0.24] to −1.18 [−1.87, −0.53].

**Table 3:** Estimated concurrent changes in Valence for 30-minute 24-h physical behavior reallocations.

	↓ Sleep period	↓ Sedentary	↓ Standing	↓ LPA	↓ MVPA
***Between-person***					
↑ Sleep period	–	−0.30 [−1.27, 0.66]	**−1.82** **[−3.32**,** −0.38]**	**−3.94** **[−7.80**,** −0.03]**	0.48 [−2.24, 3.13]
↑ Sedentary	0.34 [−0.64, 1.32]	–	**−1.51** **[−2.94**,** −0.21]**	−3.62 [−7.89, 0.44]	0.79 [−1.83, 3.35]
↑ Standing	**1.63** **[0.33**,** 2.96]**	**1.29** **[0.19**,** 2.51]**	–	−2.34 [−7.05, 2.25]	2.08 [−0.79, 4.91]
↑ LPA	**2.71** **[0.17**,** 5.19]**	2.37 [−0.30, 5.20]	0.85 [−2.44, 4.33]	–	3.16 [−1.03, 7.21]
↑ MVPA	−0.15 [−2.07, 1.78]	−0.48 [−2.29, 1.36]	−2.00 [−4.27, 0.25]	−4.11 [−8.90, 0.67]	–
***Within-person***					
↑ Sleep period	–	−0.09 [−0.29, 0.13]	−0.23 [−0.59, 0.12]	−0.71 [−1.76, 0.37]	**−1.18** **[−1.87**,** −0.53]**
↑ Sedentary	0.10 [−0.12, 0.30]	–	−0.14 [−0.47, 0.16]	−0.62 [−1.67, 0.45]	**−1.10** **[−1.77**,** −0.43]**
↑ Standing	0.22 [−0.09, 0.54]	0.13 [−0.13, 0.41]	–	−0.50 [−1.71, 0.73]	**−0.97** **[−1.73**,** −0.24]**
↑ LPA	0.52 [−0.20, 1.20]	0.42 [−0.27, 1.11]	0.28 [−0.62, 1.15]	–	−0.68 [−1.73, 0.34]
↑ MVPA	**0.86** **[0.39**,** 1.34]**	**0.77** **[0.30**,** 1.23]**	**0.62** **[0.04**,** 1.22]**	0.14 [−1.08, 1.40]	–

*Notes*. MVPA = moderate-to-vigorous physical activity, LPA = light physical activity. Values are unstandardised mean difference [unstandardised 95% credible intervals]. Bold values indicate 95% credible intervals not containing 0. Covariates included age, sex, body mass index, daily total wear time, and previous-day lag outcome.

### Energetic arousal

Reallocations of 24-h behavior were concurrently associated with energetic arousal at only the *within-person* level, with reallocations involving MVPA having the strongest associations (see Table 4; Fig. 3 panel B). +30 min more MVPA at the expense of other behaviors (sleep period, sedentary, and standing) predicted higher energetic arousal (estimates ranged from + 1.12 [0.62, 1.63] to + 1.34 [0.74, 1.95]). The opposite reallocations, that is 30 min less MVPA compensated by any other behavior was associated with lower energetic arousal; estimates ranged from − 1.22 [−2.39, −0.11] to −1.78 [−2.55, −1.02].

### Calmness

Reallocations of 24-h behavior were concurrently associated with calmness at both *between-person* and *within-person* levels (see Table 5; Fig. 2 Panel C, and Fig. 3 Panel C). At the between-person level, individuals who spent + 30-minute more standing at the expense of either sleep period, sedentary, and MVPA reported higher calmness, with estimates ranging from + 1.23 [0.04, 2.40] to + 3.01 [0.08, 5.91]. The opposite reallocations, that is 30 min less standing compensated by either sleep period, SB, or MVPA was associated with lower calmness; estimates ranged from − 1.51 [−2.86, −0.12] to −2.76 [−5.03, −0.46]. At the within-person level, 30 min more LPA relative to either sleep period or sedentary predicted higher calmness (+ 0.97[0.18, 1.81] and + 0.95 [0.18, 1.74] respectively). In contrast, 30 min less LPA compensated by any other behavior was associated with lower calmness (estimates ranged from − 1.42 [−2.83, −0.01] to −1.47 [−2.76, −0.27]).

### Prospective associations and reallocation models between 24-h physical behavior compositions and affective states

No significant associations were found between the 24-h composition and next-day affective states (see Table 2). Reallocations of 24-h behavior did not predict prospective changes in any affect types (see Supplementary for full results).

**Table 4:** Estimated concurrent changes in energetic arousal for 30-minute 24-h physical behavior reallocations.

	↓ Sleep period	↓ Sedentary	↓ Standing	↓ LPA	↓ MVPA
***Between-person***					
↑ Sleep period	–	0.29 [−0.66, 1.25]	−0.38 [−1.84, 1.08]	−3.10 [−6.96, 0.58]	1.39 [−1.27, 4.10]
↑ Sedentary	−0.28 [−1.25, 0.68]	–	−0.66 [−1.94, 0.65]	−3.38 [−7.44, 0.53]	1.11 [−1.50, 3.84]
↑ Standing	0.31 [−0.99, 1.62]	0.60 [−0.52, 1.70]	–	−2.79 [−7.23, 1.53]	1.70 [−1.26, 4.54]
↑ LPA	1.95 [−0.39, 4.42]	2.25 [−0.31, 4.91]	1.58 [−1.56, 4.80]	–	3.34 [−0.59, 7.44]
↑ MVPA	−0.96 [−2.85, 0.94]	−0.66 [−2.55, 1.15]	−1.33 [−3.55, 1.03]	−4.05 [−8.88, 0.41]	–
***Within-person***					
↑ Sleep period	–	−0.02 [−0.28, 0.23]	0.23 [−0.18, 0.62]	−0.56 [−1.79, 0.66]	**−1.61** **[−2.32**,** −0.93]**
↑ Sedentary	0.03 [−0.23, 0.29]	–	0.25 [−0.10, 0.60]	−0.53 [−1.78, 0.70]	**−1.58** **[−2.27**,** −0.92]**
↑ Standing	−0.17 [−0.52, 0.19]	−0.20 [−0.50, 0.10]	–	−0.73 [−2.15, 0.68]	**−1.78** **[−2.55**,** −1.02]**
↑ LPA	0.39 [−0.40, 1.20]	0.36 [−0.44, 1.17]	0.61 [−0.41, 1.64]	–	**−1.22** **[−2.39**,** −0.11]**
↑ MVPA	**1.12** **[0.62**,** 1.63]**	**1.10** **[0.63**,** 1.58]**	**1.34** **[0.74**,** 1.95]**	0.56 [−0.80, 1.98]	–

*Notes*. MVPA = moderate-to-vigorous physical activity, LPA = light physical activity. Values are unstandardised mean difference [unstandardised 95% credible intervals]. Bold values indicate 95% credible intervals not containing 0. Covariates included age, sex, body mass index, daily total wear time, and previous-day lag outcome.

**Table 5:** Estimated concurrent changes in calmness for 30-minute 24-h physical behavior reallocations.

	↓ Sleep period	↓ Sedentary	↓ Standing	↓ LPA	↓ MVPA
***Between-person***					
↑ Sleep period	–	−0.76 [−1.82, 0.29]	**−2.26** **[−3.82**,** −0.69]**	−2.13 [−6.10, 1.88]	1.02 [−1.81, 3.78]
↑ Sedentary	0.79 [−0.27, 1.86]	–	**−1.51** **[−2.86**,** −0.12]**	−1.37 [−5.61, 2.87]	1.77 [−0.94, 4.33]
↑ Standing	**2.03** **[0.64**,** 3.43]**	**1.23** **[0.04**,** 2.40]**	–	−0.14 [−4.79, 4.54]	**3.01** **[0.08**,** 5.91]**
↑ LPA	1.62 [−0.93, 4.13]	0.82 [−1.95, 3.68]	−0.68 [−4.09, 2.78]	–	2.60 [−1.60, 6.71]
↑ MVPA	−0.45 [−2.39, 1.58]	−1.25 [−3.03, 0.63]	**−2.76** **[−5.03**,** −0.46]**	−2.62 [−7.44, 2.25]	–
***Within-person***					
↑ Sleep period	–	−0.02 [−0.23, 0.21]	−0.05 [−0.45, 0.35]	**−1.47** **[−2.76**,** −0.27]**	−0.01 [−0.72, 0.72]
↑ Sedentary	0.02 [−0.21, 0.24]	–	−0.03 [−0.41, 0.33]	**−1.45** **[−2.70**,** −0.26]**	0.01 [−0.68, 0.71]
↑ Standing	0.06 [−0.29, 0.41]	0.04 [−0.26, 0.36]	–	**−1.42** **[−2.83**,** −0.01]**	0.04 [−0.76, 0.86]
↑ LPA	**0.97** **[0.18**,** 1.81]**	**0.95** **[0.18**,** 1.74]**	0.91 [−0.12, 1.94]	–	0.95 [−0.15, 2.11]
↑ MVPA	0.03 [−0.47, −0.47]	0.01 [−0.47, 0.49]	−0.02 [−0.67, 0.62]	**−1.44** **[−2.85**,** −0.10]**	–

*Notes*. MVPA = moderate-to-vigorous physical activity, LPA = light physical activity. Values are unstandardised mean difference [unstandardised 95% credible intervals]. Bold values indicate 95% credible intervals not containing 0. Covariates included age, sex, body mass index, daily total wear time, and previous-day lag outcome.

#### Associations and reallocation models between 24-h physical behavior compositions and working memory

In contrast to our expectations (hypothesis 1c and 2c), both concurrent and prospective analyses revealed no associations between 24-h physical behavior compositions and working memory. Reallocations were also not associated with concurrent and prospective changes in working memory (see Tables 2 and 6 for concurrent results and Supplementary for prospective results).

**Table 6:** Estimated concurrent changes in working memory for 30-minute 24-h physical behavior reallocations.

	↓ Sleep period	↓ Sedentary	↓ Standing	↓ LPA	↓ MVPA
***Between-person***					
↑ Sleep period	–	0.15 [−0.50, 0.77]	−0.26 [−1.24, 0.67]	−0.25 [−2.78, 2.28]	1.57 [−0.10, 3.27]
↑ Sedentary	−0.16 [−0.78, 0.50]	–	−0.41 [−1.27, 0.43]	−0.39 [−3.10, 2.25]	1.42 [−0.22, 3.08]
↑ Standing	0.20 [−0.64, 1.08]	0.35 [−0.36, 1.10]	–	−0.04 [−2.99, 2.91]	1.77 [−0.01, 3.59]
↑ LPA	0.11 [−1.54, 1.72]	0.27 [−1.48, 2.04]	−0.14 [−2.35, 1.96]	–	1.69 [−0.90, 4.27]
↑ MVPA	−1.11 [−2.31, 0.10]	−0.95 [−2.12, 0.19]	−1.36 [−2.80, 0.05]	−1.34 [−4.40, 1.72]	–
***Within-person***					
↑ Sleep period	–	0.09 [−0.02, 0.19]	0.02 [−0.18, 0.24]	0.48 [−0.21, 1.14]	−0.02 [−0.34, 0.31]
↑ Sedentary	−0.09 [−0.19, 0.02]	–	−0.06 [−0.25, 0.14]	0.40 [−0.27, 1.06]	−0.11 [−0.43, 0.23]
↑ Standing	−0.03 [−0.22, 0.15]	0.06 [−0.12, 0.22]	–	0.45 [−0.32, 1.20]	−0.05 [−0.43, 0.32]
↑ LPA	−0.33 [−0.76, 0.11]	−0.24 [−0.67, 0.18]	−0.30 [−0.86, 0.26]	–	−0.35 [−0.94, 0.25]
↑ MVPA	−0.01 [−0.24, 0.21]	0.08 [−0.15, 0.31]	0.02 [−0.29, 0.33]	0.48 [−0.29, 1.22]	–

*Notes*. MVPA = moderate-to-vigorous physical activity, LPA = light physical activity. Values are unstandardised mean difference [unstandardised 95% credible intervals]. Bold values indicate 95% credible intervals not containing 0. Covariates included age, sex, body mass index, daily total wear time, and previous-day lag outcome.

## Discussion

This study aimed to gain insights into the compositional effects of 24-h physical behaviors, affective states (valence, energetic arousal, and calmness), and working memory performance in the daily life of working adults. As one of the first studies, we captured device-based measurements of five physical behaviors within the 24-h Activity Cycle (i.e., MVPA, LPA, standing, SB, and sleep period) and multiple daily ratings of affective states and working memory performance via smartphone. Using Bayesian multilevel compositional data analysis, we observed that more time spent on MVPA relative to the remaining behaviors was associated with same-day higher valence and energetic arousal (but not calmness) at the within-person, not between-person level. In contrast to our expectations, SB in relation to remaining behaviors was not associated with any affective states. Further, more time in MVPA, followed by LPA, and standing at the expense of other behaviors (SB and sleep period) was associated with better affective outcomes. These associations emerged only concurrently (same day) but not prospectively (next day), which support the short-term mechanism underlying the association between behaviours and affect. Our results suggest that more MVPA relative to the remaining behaviors is beneficial for two of three affective states (i.e., valence and energetic arousal), whereas more MVPA at the expense of some, but not all, other behaviors are negatively associated with calmness. These findings are in line with previous studies using compositional analysis, showing the positive impact of MVPA on affective states^55–57^. For example, on a daily level, more MVPA at the expense of LPA or SB was associated with higher high arousal positive affect^57^, replacing MVPA with SB predicted higher valence^55^, and a higher ratio of MVPA to SB and LPA predicted higher both valence and energetic arousal within a 60-minute timeframe^36^.

Physiological mechanisms suggest that MVPA may lead to improvements in cortical structures (e.g., prefontal or motor cortex) involved in regulating affective states^58^. Moreover, affect has been found to be especially positive and higher during high-intensity interval training, emphasizing the significance of intensity^59^. However, there are less consistent data available that provide causal insights into the physiological mechanism linking MVPA to affective states. Above all, it is a methodological challenge to collect physiological parameters continuously in daily life. Schenk et al. showed that the assessment of urinary inflammatory markers is feasible in an intensive day-to-day study in healthy individuals but is still limited to providing continuous information^60^. A first indirect approach might be to collect data via continuous glucose monitoring^61^, since inflammatory markers can affect metabolism and have an impact on blood sugar levels^62^.

Reallocations involving LPA were mostly associated with changes in calmness, particularly when it is increased at the expense of SB. This supports evidence that even short bouts of LPA have positive effects on affective states^31,56^. In particular, previous studies have shown that LPA is associated with momentary increased positive affect^63^, increased energetic arousal, and increased calmness^64^. Giurgiu et al. (2020) have also shown that breaking-up SB with an intensity of 1.6 METs (e.g., standing), enhanced valence by 8.29 points on a scale from 0 to 100, and activities with higher intensities (3.5 METs), such as moderate walking, enhanced valence by 18.13 points on average^30^. Our finding extends previous evidence by showing the positive associations between LPA and affect occurred when it was increased at the expense of SB. This finding suggests that short sedentary breaks with lower intensity activities (e.g., standing, slow break) might be helpful to regulate momentary affective states in daily life. Emerging studies support this finding, showing significant improvements in psychological outcomes, such as mood, by using a sit-stand desk in office workers^65^. Future research may implement ambulatory assessment techniques in personalized just-in-time adaptive interventions, as they can promote short walking breaks through triggered prompts in daily life^66^.

Previous studies highlighted the negative impact of SB on affective states, especially on valence and energetic arousal^31,56,67^. In contrast to our hypotheses, SB relative to the other behaviors was not associated with energetic arousal, calmness, valence, and working memory. One methodological explanation might be that our sedentary triggered design (i.e., half of the assessments were captured after a sedentary bout of ≥ 30 min) could result in lower daily affect ratings. However, our reallocation approach revealed that shifting time from MVPA to SB was significantly associated with poorer valence and energetic arousal, suggesting that the negative association between SB and affect emerge particularly when MVPA was compensated as a result. This finding suggests that the affective impacts could be due to the combination of prolonged SB and reduced MVPA, highlighting the co-dependence between these behaviours.

Less time in sleep when compensated by other 24-h behaviors, including MVPA, LPA, and standing showed positive effects on all types of affects. This finding differed from previous compositional analysis showing that longer sleep duration was cross-sectionally associated with lower high arousal negative affect Le et al. (2022). This previous study and the existing sleep-affect literature^68^support the association between poor sleep and worse affect. Several conceptual and methodological differences across studies might explain these discrepant findings; we propose the following. The current study examined sleep period (i.e., time in bed) which encompasses both sleep duration (time in bed actually sleeping) and time awake in bed (time taken to fall asleep in bed plus awakening throughout the night), whereas Le et al. (2022) examined these two sleep parameters separately. Bed extension (extended time in bed) might involve SB, which is associated with elevated depressive symptoms^69^. Thus, our participants might have engaged in SB during their sleep period, which might explain the similar patterns of associations for sleep and sedentary reallocations found in our study. Empirical evidence also link that sleep duration to better affect, whereas time awake in bed to worse affect^70^; this was observed in Le et al. (2022)’s analysis. Future studies should include both sleep and time awake in bed to examine their separatable associations with affect. Additionally, the significant results between sleep and affect emerged from affect measured prior to sleep recording in the evening. Thus, reverse causations are plausible, such that worse affect during the day leads to poor sleep that night.

Findings only emerged at the same-day, concurrent level, but not next day, prospective level support the short-term, within-person fluctuation of both 24-h physical behaviours and affect. They are consistent with previous studies which found that effect of MVPA on affect remained within 8-hour time interval before starting to dissipate^71^. The weak magnitudes of within-person associations is consistent with previous analyses at the daily level and among health individuals^31,72^. However, these small daily effects might accumulate over time and result in more profound associations. Longitudinal studies capturing behaviours and affect over longer period of time are needed to provide further insights into such cumulative effects. The concurrent associatons might also reflect reversed causation bias (affect predicting behaviours); future analyses could examine the temporal dynamics with behaviours preceding affect to provide more robust test of directionality.

Physical behaviors were not associated with working memory. To our knowledge, behavior-induced changes in cognitive health remain largely understudied and show controversial findings^18^. A recent review reflects the inconclusive state of research, indicating that only in certain cases have short bouts of physical activity interrupting prolonged sedentary time been shown to positively impact cognitive function. So far, only a few studies have examined the associations between 24-h physical behaviors and cognitive functions^18^. For example, a recent study has shown that additional 30 min of MVPA on the previous day was associated with episodic memory scores which may persist for 24 h^73^. Moreover, sleep duration ≥ 6 h (compared with < 6 h) on the previous night was associated with episodic memory scores^73^. Our assessment of working memory performance extended previous methods by using a mobile cognitive task in real-time and under real-life conditions by integrating up to six tests per day. Although the number of existing studies that used mobile cognitive tasks is only a few, the first reliability and validity results concluded that brief cognitive assessments made in uncontrolled naturalistic settings provide measurements that are comparable in reliability to assessments made in controlled laboratory environments^74^. Another explanation might be that 24-h physical behaviors are more related to other executive functions such as inhibition control or mental flexibility than to working memory performance.

Several limitations warrant further discussion. We included only healthy university employees, therefore findings may not generalize to other populations. For instance, our recruited sample showed a higher level of MVPA per day compared to similar study samples^75^. Further studies should incorporate more diverse samples, especially individuals with mental or sleep disorders, where stronger associations between 24-h behaviors, affective states, and working memory might be observed. Studies with larger size and more diverse sample could allow for the potential non-linear associations between 24-h behaviours and affect to be captured, given both sleep and physical activity have demonstrated non-linear association with affect^71,76^. Our concurrent analysis did not establish temporal order and directionality of the associations, thus could be biased by reverse causation. Future experimental studies in naturalistic settings are needed to establish causality. The numerical updating task to measure working memory performance has shown ceiling effects. To better understand the influence of 24-h behaviors on executive functions, more outcomes of cognitive function and adaptive versions of measurements are needed (e.g., changing the test specification based on the previous results). Future research should at least control potential learning effects through repeated measurements of mobile cognitive tasks. Further, in half of all trigger points, outcome measures were captured after a sedentary bout (i.e., 30 min uninterrupted sitting/lying), which may reduce variability and could be a source of bias for lower ratings. Therefore, future studies should integrate random time points (e.g., providing a mixed-sampling scheme). We also designed weekend days as break days, consequently, the assessments might be biased towards a reduced proportion of leisure time PA. We did not adjust for multiple comparisons due to the current lack of adjustment methods in Bayesian analysis; some of the intervals might include zero if stringent intervals were used. Lastly, the used sleep algorithm may misclassify daytime sleep as SB (i.e., if not a primary time-in-bed period was detected^44^). However, since in only 5.7% of all analyzed days, participants reported daytime sleep via e-diaries in the evening, this might be a minor issue for the analyses.

In conclusion, our analysis underscores the importance of a 24-h approach in the context of mental health. In the context of a 24-h day, MVPA, followed by LPA, and standing, at the expense of other behaviors (SB and sleep period) was associated with better affective outcomes. The same-day associations between 24-h behavior composition and affective states, but not prospective associations found in our study reflect the short-term, within-person interaction between behaviours and affect. Reallocating time from one behavior to another is generally associated with affective states at both the within-person and the between-person levels. Future studies may benefit from investigating associations with a broader range of mental health outcomes and using more diverse samples, particularly individuals with mental disorders. More research, especially intervention and experimental studies in naturalistic settings, using digital technologies with ambulatory assessment is needed to draw further conclusions about associations between 24-h behavior composition and mental health outcomes across different populations and furthermore to advance 24-h guidelines with regard to a holistic health approach.

## Electronic supplementary material

Below is the link to the electronic supplementary material.

Supplementary Material 1

## Data Availability

The datasets used and/or analysed during the current study are available from the corresponding author upon reasonable request.

## Abbreviations

SB: Sedentary behavior
PA: physical activity
MVPA: Moderate-to-vigorous physical activity
LPA: Light physical activity
CoDA: Compositional data analyses

## Declarations

### Patient and public involvement

Patients and/or the public were not involved in the design, conduct, reporting or dissemination plans of this research.
