# Supplementary material for: Associations between daily composition of 24 h physical behavior with affective states and working memory
Source: Sci Rep. 2025 Apr 25;15:14455. doi: 10.1038/s41598-025-99266-4 (PMC12032417; doi:10.1038/s41598-025-99266-4)
Supplement: Supplementary file 1 — Supplementary Material 1 [file 41598_2025_99266_MOESM1_ESM.html]

Supplementary materials: Sensitivity analysis for Associations between daily composition of 24-hour physical behavior with affective states and working memory


# Supplementary materials: Sensitivity analysis for Associations between daily composition of 24-hour physical behavior with affective states and working memory

#### Flora Le (flora.le@monash.edu)

#### 2025-03-26

This file presents the full results for the analysis of
**Associations between daily composition of 24-hour physical
behavior with affective states and working memory**.

*Notes.* Statistical significance of individual parameters was
set as Bayesian 95% posterior credible intervals (CIs) not including
zero. Sleep = Sleep Period. MVPA = Moderate-to-Vigorous Physical
Activity. LPA = Light Physical Activity.

# 1 Sensitivity results using at least 3 ratings of affect per day

## 1.1 60-minute reallocations

- Reallocation of 60 minutes between movement behaviours and
  *valence*

| Estimate [95% CI] | Minute | From | To | Level | Sig |
| --- | --- | --- | --- | --- | --- |
| -0.59 [-2.57, 1.41] | 60 | Sedentary | Sleep | between |  |
| -3.47 [-7.13, 0.03] | 60 | Standing | Sleep | between |  |
| -10.67 [-25.1, 3.29] | 60 | LPA | Sleep | between |  |
| 2.28 [-5.36, 9.68] | 60 | MVPA | Sleep | between |  |
| 0.71 [-1.37, 2.76] | 60 | Sleep | Sedentary | between |  |
| -2.87 [-6.01, 0.35] | 60 | Standing | Sedentary | between |  |
| -10.07 [-25.1, 4.59] | 60 | LPA | Sedentary | between |  |
| 2.88 [-4.74, 10.26] | 60 | MVPA | Sedentary | between |  |
| 2.71 [0.04, 5.49] | 60 | Sleep | Standing | between | Yes |
| 2.02 [-0.29, 4.29] | 60 | Sedentary | Standing | between |  |
| -8.07 [-24.04, 7.16] | 60 | LPA | Standing | between |  |
| 4.88 [-3.48, 12.84] | 60 | MVPA | Standing | between |  |
| 3.84 [-0.31, 8.15] | 60 | Sleep | LPA | between |  |
| 3.15 [-1.72, 8.01] | 60 | Sedentary | LPA | between |  |
| 0.27 [-6.26, 6.92] | 60 | Standing | LPA | between |  |
| 6.02 [-2.9, 15.27] | 60 | MVPA | LPA | between |  |
| -0.33 [-3.61, 2.9] | 60 | Sleep | MVPA | between |  |
| -1.02 [-4.16, 2.24] | 60 | Sedentary | MVPA | between |  |
| -3.9 [-8.63, 1.08] | 60 | Standing | MVPA | between |  |
| -11.11 [-26.61, 3.64] | 60 | LPA | MVPA | between |  |
| -0.32 [-0.78, 0.13] | 60 | Sedentary | Sleep | within |  |
| -0.96 [-1.91, 0.03] | 60 | Standing | Sleep | within |  |
| -0.67 [-4.97, 3.39] | 60 | LPA | Sleep | within |  |
| -3.5 [-5.48, -1.59] | 60 | MVPA | Sleep | within | Yes |
| 0.38 [-0.1, 0.85] | 60 | Sleep | Sedentary | within |  |
| -0.63 [-1.46, 0.21] | 60 | Standing | Sedentary | within |  |
| -0.34 [-4.63, 3.66] | 60 | LPA | Sedentary | within |  |
| -3.17 [-5.13, -1.33] | 60 | MVPA | Sedentary | within | Yes |
| 0.82 [0.07, 1.54] | 60 | Sleep | Standing | within | Yes |
| 0.45 [-0.14, 1.03] | 60 | Sedentary | Standing | within |  |
| 0.1 [-4.62, 4.52] | 60 | LPA | Standing | within |  |
| -2.73 [-4.83, -0.71] | 60 | MVPA | Standing | within | Yes |
| 0.51 [-0.78, 1.87] | 60 | Sleep | LPA | within |  |
| 0.14 [-1.11, 1.46] | 60 | Sedentary | LPA | within |  |
| -0.5 [-2.35, 1.42] | 60 | Standing | LPA | within |  |
| -3.04 [-5.55, -0.59] | 60 | MVPA | LPA | within | Yes |
| 1.66 [0.81, 2.53] | 60 | Sleep | MVPA | within | Yes |
| 1.29 [0.52, 2.1] | 60 | Sedentary | MVPA | within | Yes |
| 0.65 [-0.53, 1.85] | 60 | Standing | MVPA | within |  |
| 0.94 [-3.56, 5.19] | 60 | LPA | MVPA | within |  |

- Reallocation of 60 minutes between movement behaviours and
  *energetic arousal*

| Estimate [95% CI] | Minute | From | To | Level | Sig |
| --- | --- | --- | --- | --- | --- |
| 0.36 [-1.51, 2.28] | 60 | Sedentary | Sleep | between |  |
| -0.74 [-4.22, 2.73] | 60 | Standing | Sleep | between |  |
| -7.35 [-21.15, 6.19] | 60 | LPA | Sleep | between |  |
| 5.92 [-1.35, 13.73] | 60 | MVPA | Sleep | between |  |
| -0.35 [-2.32, 1.57] | 60 | Sleep | Sedentary | between |  |
| -1.07 [-4.16, 1.99] | 60 | Standing | Sedentary | between |  |
| -7.69 [-22.02, 6.44] | 60 | LPA | Sedentary | between |  |
| 5.59 [-1.46, 13.05] | 60 | MVPA | Sedentary | between |  |
| 0.45 [-2.18, 3.08] | 60 | Sleep | Standing | between |  |
| 0.82 [-1.4, 3] | 60 | Sedentary | Standing | between |  |
| -6.89 [-21.83, 8.09] | 60 | LPA | Standing | between |  |
| 6.39 [-1.34, 14.38] | 60 | MVPA | Standing | between |  |
| 2.08 [-1.85, 6.13] | 60 | Sleep | LPA | between |  |
| 2.46 [-2.12, 7.1] | 60 | Sedentary | LPA | between |  |
| 1.36 [-4.84, 7.58] | 60 | Standing | LPA | between |  |
| 8.02 [-0.57, 17.56] | 60 | MVPA | LPA | between |  |
| -2.42 [-5.73, 0.8] | 60 | Sleep | MVPA | between |  |
| -2.04 [-5.11, 0.9] | 60 | Sedentary | MVPA | between |  |
| -3.14 [-7.81, 1.37] | 60 | Standing | MVPA | between |  |
| -9.75 [-24.58, 4.91] | 60 | LPA | MVPA | between |  |
| -0.12 [-0.63, 0.38] | 60 | Sedentary | Sleep | within |  |
| 0.26 [-0.69, 1.22] | 60 | Standing | Sleep | within |  |
| -0.53 [-5.2, 4.28] | 60 | LPA | Sleep | within |  |
| -4.54 [-6.62, -2.44] | 60 | MVPA | Sleep | within | Yes |
| 0.15 [-0.37, 0.68] | 60 | Sleep | Sedentary | within |  |
| 0.38 [-0.49, 1.25] | 60 | Standing | Sedentary | within |  |
| -0.41 [-5.05, 4.37] | 60 | LPA | Sedentary | within |  |
| -4.41 [-6.48, -2.35] | 60 | MVPA | Sedentary | within | Yes |
| -0.1 [-0.81, 0.63] | 60 | Sleep | Standing | within |  |
| -0.24 [-0.84, 0.38] | 60 | Sedentary | Standing | within |  |
| -0.65 [-5.63, 4.48] | 60 | LPA | Standing | within |  |
| -4.66 [-6.87, -2.47] | 60 | MVPA | Standing | within | Yes |
| 0.31 [-1.16, 1.75] | 60 | Sleep | LPA | within |  |
| 0.17 [-1.28, 1.61] | 60 | Sedentary | LPA | within |  |
| 0.55 [-1.45, 2.59] | 60 | Standing | LPA | within |  |
| -4.25 [-7.02, -1.49] | 60 | MVPA | LPA | within | Yes |
| 1.91 [0.99, 2.83] | 60 | Sleep | MVPA | within | Yes |
| 1.77 [0.92, 2.65] | 60 | Sedentary | MVPA | within | Yes |
| 2.15 [0.91, 3.43] | 60 | Standing | MVPA | within | Yes |
| 1.36 [-3.58, 6.31] | 60 | LPA | MVPA | within |  |

- Reallocation of 60 minutes between movement behaviours and
  *calmness*

| Estimate [95% CI] | Minute | From | To | Level | Sig |
| --- | --- | --- | --- | --- | --- |
| -1.08 [-3.03, 0.84] | 60 | Sedentary | Sleep | between |  |
| -5.23 [-8.82, -1.7] | 60 | Standing | Sleep | between | Yes |
| -8.23 [-22.95, 5.97] | 60 | LPA | Sleep | between |  |
| 4.03 [-3.17, 11] | 60 | MVPA | Sleep | between |  |
| 1.22 [-0.74, 3.23] | 60 | Sleep | Sedentary | between |  |
| -4.15 [-7.29, -0.94] | 60 | Standing | Sedentary | between | Yes |
| -7.15 [-22.66, 7.6] | 60 | LPA | Sedentary | between |  |
| 5.11 [-2.17, 11.99] | 60 | MVPA | Sedentary | between |  |
| 4.02 [1.35, 6.71] | 60 | Sleep | Standing | between | Yes |
| 2.8 [0.5, 5.01] | 60 | Sedentary | Standing | between | Yes |
| -4.35 [-20.53, 10.98] | 60 | LPA | Standing | between |  |
| 7.91 [0.35, 15.23] | 60 | MVPA | Standing | between | Yes |
| 3.32 [-0.89, 7.68] | 60 | Sleep | LPA | between |  |
| 2.1 [-2.72, 7.23] | 60 | Sedentary | LPA | between |  |
| -2.05 [-8.67, 4.44] | 60 | Standing | LPA | between |  |
| 7.21 [-1.79, 15.83] | 60 | MVPA | LPA | between |  |
| -0.87 [-4.03, 2.39] | 60 | Sleep | MVPA | between |  |
| -2.1 [-5.05, 1.08] | 60 | Sedentary | MVPA | between |  |
| -6.25 [-10.86, -1.81] | 60 | Standing | MVPA | between | Yes |
| -9.24 [-24.9, 5.94] | 60 | LPA | MVPA | between |  |
| 0.01 [-0.4, 0.4] | 60 | Sedentary | Sleep | within |  |
| -0.13 [-1.07, 0.79] | 60 | Standing | Sleep | within |  |
| -5.15 [-9.75, -0.62] | 60 | LPA | Sleep | within | Yes |
| 0.52 [-1.44, 2.44] | 60 | MVPA | Sleep | within |  |
| 0.02 [-0.39, 0.44] | 60 | Sleep | Sedentary | within |  |
| -0.13 [-1, 0.77] | 60 | Standing | Sedentary | within |  |
| -5.14 [-9.67, -0.64] | 60 | LPA | Sedentary | within | Yes |
| 0.53 [-1.42, 2.4] | 60 | MVPA | Sedentary | within |  |
| 0.14 [-0.53, 0.82] | 60 | Sleep | Standing | within |  |
| 0.13 [-0.5, 0.74] | 60 | Sedentary | Standing | within |  |
| -5.02 [-9.9, -0.17] | 60 | LPA | Standing | within | Yes |
| 0.64 [-1.44, 2.71] | 60 | MVPA | Standing | within |  |
| 1.62 [0.21, 3.05] | 60 | Sleep | LPA | within | Yes |
| 1.61 [0.24, 2.99] | 60 | Sedentary | LPA | within | Yes |
| 1.47 [-0.48, 3.44] | 60 | Standing | LPA | within |  |
| 2.12 [-0.44, 4.68] | 60 | MVPA | LPA | within |  |
| -0.13 [-0.92, 0.71] | 60 | Sleep | MVPA | within |  |
| -0.14 [-0.9, 0.66] | 60 | Sedentary | MVPA | within |  |
| -0.27 [-1.5, 0.95] | 60 | Standing | MVPA | within |  |
| -5.29 [-10.04, -0.6] | 60 | LPA | MVPA | within | Yes |

- Reallocation of 60 minutes between movement behaviours and
  *working memory performance*

| Estimate [95% CI] | Minute | From | To | Level | Sig |
| --- | --- | --- | --- | --- | --- |
| -0.11 [-1.39, 1.16] | 60 | Sedentary | Sleep | between |  |
| -1.59 [-3.77, 0.67] | 60 | Standing | Sleep | between |  |
| -3.57 [-13.28, 6.44] | 60 | LPA | Sleep | between |  |
| 3.92 [-1.06, 8.86] | 60 | MVPA | Sleep | between |  |
| 0.14 [-1.17, 1.44] | 60 | Sleep | Sedentary | between |  |
| -1.47 [-3.51, 0.57] | 60 | Standing | Sedentary | between |  |
| -3.45 [-13.51, 6.93] | 60 | LPA | Sedentary | between |  |
| 4.04 [-0.79, 8.83] | 60 | MVPA | Sedentary | between |  |
| 1.15 [-0.54, 2.77] | 60 | Sleep | Standing | between |  |
| 1.02 [-0.44, 2.51] | 60 | Sedentary | Standing | between |  |
| -2.44 [-13, 8.42] | 60 | LPA | Standing | between |  |
| 5.05 [-0.15, 10.3] | 60 | MVPA | Standing | between |  |
| 1.2 [-1.74, 4] | 60 | Sleep | LPA | between |  |
| 1.06 [-2.27, 4.26] | 60 | Sedentary | LPA | between |  |
| -0.41 [-4.79, 4.03] | 60 | Standing | LPA | between |  |
| 5.09 [-1.13, 11.17] | 60 | MVPA | LPA | between |  |
| -1.42 [-3.56, 0.72] | 60 | Sleep | MVPA | between |  |
| -1.55 [-3.53, 0.44] | 60 | Sedentary | MVPA | between |  |
| -3.03 [-6.04, -0.03] | 60 | Standing | MVPA | between | Yes |
| -5.01 [-15.33, 5.62] | 60 | LPA | MVPA | between |  |
| 0.23 [-0.03, 0.48] | 60 | Sedentary | Sleep | within |  |
| 0.22 [-0.44, 0.92] | 60 | Standing | Sleep | within |  |
| 1.67 [-1.65, 5.06] | 60 | LPA | Sleep | within |  |
| -0.39 [-1.52, 0.72] | 60 | MVPA | Sleep | within |  |
| -0.24 [-0.51, 0.02] | 60 | Sleep | Sedentary | within |  |
| 0 [-0.64, 0.7] | 60 | Standing | Sedentary | within |  |
| 1.45 [-1.87, 4.81] | 60 | LPA | Sedentary | within |  |
| -0.61 [-1.73, 0.5] | 60 | MVPA | Sedentary | within |  |
| -0.22 [-0.72, 0.26] | 60 | Sleep | Standing | within |  |
| 0.03 [-0.46, 0.48] | 60 | Sedentary | Standing | within |  |
| 1.47 [-2.19, 5.1] | 60 | LPA | Standing | within |  |
| -0.59 [-1.83, 0.61] | 60 | MVPA | Standing | within |  |
| -0.62 [-1.67, 0.41] | 60 | Sleep | LPA | within |  |
| -0.38 [-1.4, 0.63] | 60 | Sedentary | LPA | within |  |
| -0.38 [-1.86, 1.15] | 60 | Standing | LPA | within |  |
| -0.99 [-2.59, 0.62] | 60 | MVPA | LPA | within |  |
| 0.04 [-0.43, 0.53] | 60 | Sleep | MVPA | within |  |
| 0.29 [-0.17, 0.76] | 60 | Sedentary | MVPA | within |  |
| 0.28 [-0.53, 1.12] | 60 | Standing | MVPA | within |  |
| 1.73 [-1.74, 5.22] | 60 | LPA | MVPA | within |  |

## 1.2 30-minute reallocations

- Reallocation of 30 minutes between movement behaviours and
  *valence*

| Estimate [95% CI] | Minute | From | To | Level | Sig |
| --- | --- | --- | --- | --- | --- |
| -0.31 [-1.3, 0.7] | 30 | Sedentary | Sleep | between |  |
| -1.59 [-3.26, 0.01] | 30 | Standing | Sleep | between |  |
| -3.12 [-7.11, 0.66] | 30 | LPA | Sleep | between |  |
| 0.62 [-1.97, 3.14] | 30 | MVPA | Sleep | between |  |
| 0.34 [-0.69, 1.35] | 30 | Sleep | Sedentary | between |  |
| -1.28 [-2.69, 0.16] | 30 | Standing | Sedentary | between |  |
| -2.81 [-7.01, 1.35] | 30 | LPA | Sedentary | between |  |
| 0.93 [-1.68, 3.45] | 30 | MVPA | Sedentary | between |  |
| 1.41 [0.01, 2.87] | 30 | Sleep | Standing | between | Yes |
| 1.08 [-0.14, 2.29] | 30 | Sedentary | Standing | between |  |
| -1.73 [-6.45, 2.83] | 30 | LPA | Standing | between |  |
| 2.01 [-1.07, 4.92] | 30 | MVPA | Standing | between |  |
| 2.14 [-0.25, 4.63] | 30 | Sleep | LPA | between |  |
| 1.8 [-0.94, 4.56] | 30 | Sedentary | LPA | between |  |
| 0.52 [-2.89, 3.98] | 30 | Standing | LPA | between |  |
| 2.73 [-0.9, 6.54] | 30 | MVPA | LPA | between |  |
| -0.26 [-2.08, 1.53] | 30 | Sleep | MVPA | between |  |
| -0.6 [-2.36, 1.21] | 30 | Sedentary | MVPA | between |  |
| -1.88 [-4.23, 0.65] | 30 | Standing | MVPA | between |  |
| -3.41 [-8.03, 0.92] | 30 | LPA | MVPA | between |  |
| -0.17 [-0.4, 0.06] | 30 | Sedentary | Sleep | within |  |
| -0.45 [-0.89, 0] | 30 | Standing | Sleep | within |  |
| -0.27 [-1.47, 0.86] | 30 | LPA | Sleep | within |  |
| -1.24 [-1.92, -0.58] | 30 | MVPA | Sleep | within | Yes |
| 0.18 [-0.05, 0.41] | 30 | Sleep | Sedentary | within |  |
| -0.28 [-0.65, 0.09] | 30 | Standing | Sedentary | within |  |
| -0.1 [-1.31, 1.01] | 30 | LPA | Sedentary | within |  |
| -1.07 [-1.73, -0.44] | 30 | MVPA | Sedentary | within | Yes |
| 0.42 [0.03, 0.8] | 30 | Sleep | Standing | within | Yes |
| 0.24 [-0.07, 0.55] | 30 | Sedentary | Standing | within |  |
| 0.13 [-1.28, 1.45] | 30 | LPA | Standing | within |  |
| -0.83 [-1.59, -0.11] | 30 | MVPA | Standing | within | Yes |
| 0.25 [-0.48, 1.03] | 30 | Sleep | LPA | within |  |
| 0.07 [-0.64, 0.84] | 30 | Sedentary | LPA | within |  |
| -0.21 [-1.18, 0.8] | 30 | Standing | LPA | within |  |
| -1 [-2.05, 0.06] | 30 | MVPA | LPA | within |  |
| 0.91 [0.44, 1.39] | 30 | Sleep | MVPA | within | Yes |
| 0.73 [0.3, 1.18] | 30 | Sedentary | MVPA | within | Yes |
| 0.45 [-0.15, 1.05] | 30 | Standing | MVPA | within |  |
| 0.62 [-0.7, 1.9] | 30 | LPA | MVPA | within |  |

- Reallocation of 30 minutes between movement behaviours and
  *energetic arousal*

| Estimate [95% CI] | Minute | From | To | Level | Sig |
| --- | --- | --- | --- | --- | --- |
| 0.18 [-0.76, 1.14] | 30 | Sedentary | Sleep | between |  |
| -0.32 [-1.89, 1.25] | 30 | Standing | Sleep | between |  |
| -1.98 [-5.71, 1.7] | 30 | LPA | Sleep | between |  |
| 1.99 [-0.52, 4.63] | 30 | MVPA | Sleep | between |  |
| -0.18 [-1.15, 0.78] | 30 | Sleep | Sedentary | between |  |
| -0.49 [-1.86, 0.89] | 30 | Standing | Sedentary | between |  |
| -2.15 [-6.17, 1.82] | 30 | LPA | Sedentary | between |  |
| 1.82 [-0.56, 4.33] | 30 | MVPA | Sedentary | between |  |
| 0.25 [-1.12, 1.63] | 30 | Sleep | Standing | between |  |
| 0.43 [-0.76, 1.59] | 30 | Sedentary | Standing | between |  |
| -1.73 [-6.19, 2.69] | 30 | LPA | Standing | between |  |
| 2.25 [-0.49, 5.14] | 30 | MVPA | Standing | between |  |
| 1.22 [-1.06, 3.55] | 30 | Sleep | LPA | between |  |
| 1.4 [-1.2, 4.02] | 30 | Sedentary | LPA | between |  |
| 0.9 [-2.34, 4.16] | 30 | Standing | LPA | between |  |
| 3.22 [-0.36, 7.12] | 30 | MVPA | LPA | between |  |
| -1.37 [-3.22, 0.4] | 30 | Sleep | MVPA | between |  |
| -1.18 [-2.89, 0.47] | 30 | Sedentary | MVPA | between |  |
| -1.68 [-4.03, 0.59] | 30 | Standing | MVPA | between |  |
| -3.34 [-7.93, 1.07] | 30 | LPA | MVPA | between |  |
| -0.06 [-0.32, 0.19] | 30 | Sedentary | Sleep | within |  |
| 0.1 [-0.33, 0.54] | 30 | Standing | Sleep | within |  |
| -0.19 [-1.46, 1.12] | 30 | LPA | Sleep | within |  |
| -1.54 [-2.26, -0.82] | 30 | MVPA | Sleep | within | Yes |
| 0.07 [-0.19, 0.33] | 30 | Sleep | Sedentary | within |  |
| 0.17 [-0.22, 0.55] | 30 | Standing | Sedentary | within |  |
| -0.13 [-1.4, 1.18] | 30 | LPA | Sedentary | within |  |
| -1.48 [-2.19, -0.78] | 30 | MVPA | Sedentary | within | Yes |
| -0.06 [-0.44, 0.32] | 30 | Sleep | Standing | within |  |
| -0.13 [-0.45, 0.2] | 30 | Sedentary | Standing | within |  |
| -0.26 [-1.75, 1.23] | 30 | LPA | Standing | within |  |
| -1.61 [-2.4, -0.83] | 30 | MVPA | Standing | within | Yes |
| 0.16 [-0.68, 0.98] | 30 | Sleep | LPA | within |  |
| 0.09 [-0.74, 0.91] | 30 | Sedentary | LPA | within |  |
| 0.26 [-0.8, 1.35] | 30 | Standing | LPA | within |  |
| -1.39 [-2.57, -0.21] | 30 | MVPA | LPA | within | Yes |
| 1.07 [0.56, 1.58] | 30 | Sleep | MVPA | within | Yes |
| 1.01 [0.52, 1.49] | 30 | Sedentary | MVPA | within | Yes |
| 1.17 [0.55, 1.82] | 30 | Standing | MVPA | within | Yes |
| 0.88 [-0.57, 2.36] | 30 | LPA | MVPA | within |  |

- Reallocation of 30 minutes between movement behaviours and
  *calmness*

| Estimate [95% CI] | Minute | From | To | Level | Sig |
| --- | --- | --- | --- | --- | --- |
| -0.56 [-1.53, 0.41] | 30 | Sedentary | Sleep | between |  |
| -2.39 [-4.01, -0.78] | 30 | Standing | Sleep | between | Yes |
| -2.5 [-6.48, 1.36] | 30 | LPA | Sleep | between |  |
| 1.19 [-1.34, 3.62] | 30 | MVPA | Sleep | between |  |
| 0.59 [-0.38, 1.59] | 30 | Sleep | Sedentary | between |  |
| -1.83 [-3.24, -0.4] | 30 | Standing | Sedentary | between | Yes |
| -1.94 [-6.36, 2.17] | 30 | LPA | Sedentary | between |  |
| 1.74 [-0.78, 4.14] | 30 | MVPA | Sedentary | between |  |
| 2.1 [0.7, 3.52] | 30 | Sleep | Standing | between | Yes |
| 1.51 [0.3, 2.69] | 30 | Sedentary | Standing | between | Yes |
| -0.43 [-5.22, 4.15] | 30 | LPA | Standing | between |  |
| 3.26 [0.57, 6.04] | 30 | MVPA | Standing | between | Yes |
| 1.81 [-0.62, 4.33] | 30 | Sleep | LPA | between |  |
| 1.22 [-1.49, 4.11] | 30 | Sedentary | LPA | between |  |
| -0.62 [-4.01, 2.83] | 30 | Standing | LPA | between |  |
| 2.96 [-0.8, 6.65] | 30 | MVPA | LPA | between |  |
| -0.6 [-2.36, 1.18] | 30 | Sleep | MVPA | between |  |
| -1.19 [-2.86, 0.58] | 30 | Sedentary | MVPA | between |  |
| -3.02 [-5.33, -0.8] | 30 | Standing | MVPA | between | Yes |
| -3.13 [-7.65, 1.34] | 30 | LPA | MVPA | between |  |
| 0 [-0.2, 0.2] | 30 | Sedentary | Sleep | within |  |
| -0.07 [-0.49, 0.34] | 30 | Standing | Sleep | within |  |
| -1.43 [-2.7, -0.18] | 30 | LPA | Sleep | within | Yes |
| 0.15 [-0.51, 0.8] | 30 | MVPA | Sleep | within |  |
| 0.01 [-0.2, 0.21] | 30 | Sleep | Sedentary | within |  |
| -0.06 [-0.45, 0.34] | 30 | Standing | Sedentary | within |  |
| -1.43 [-2.67, -0.19] | 30 | LPA | Sedentary | within | Yes |
| 0.16 [-0.5, 0.79] | 30 | MVPA | Sedentary | within |  |
| 0.07 [-0.28, 0.43] | 30 | Sleep | Standing | within |  |
| 0.06 [-0.27, 0.39] | 30 | Sedentary | Standing | within |  |
| -1.37 [-2.81, 0.07] | 30 | LPA | Standing | within |  |
| 0.22 [-0.54, 0.97] | 30 | MVPA | Standing | within |  |
| 0.92 [0.11, 1.74] | 30 | Sleep | LPA | within | Yes |
| 0.92 [0.13, 1.71] | 30 | Sedentary | LPA | within | Yes |
| 0.85 [-0.18, 1.9] | 30 | Standing | LPA | within |  |
| 1.07 [-0.04, 2.19] | 30 | MVPA | LPA | within |  |
| -0.08 [-0.53, 0.38] | 30 | Sleep | MVPA | within |  |
| -0.09 [-0.52, 0.36] | 30 | Sedentary | MVPA | within |  |
| -0.15 [-0.76, 0.47] | 30 | Standing | MVPA | within |  |
| -1.52 [-2.93, -0.16] | 30 | LPA | MVPA | within | Yes |

- Reallocation of 30 minutes between movement behaviours and
  *working memory performance*

| Estimate [95% CI] | Minute | From | To | Level | Sig |
| --- | --- | --- | --- | --- | --- |
| -0.06 [-0.7, 0.58] | 30 | Sedentary | Sleep | between |  |
| -0.71 [-1.69, 0.31] | 30 | Standing | Sleep | between |  |
| -1.01 [-3.65, 1.72] | 30 | LPA | Sleep | between |  |
| 1.28 [-0.42, 2.96] | 30 | MVPA | Sleep | between |  |
| 0.07 [-0.58, 0.71] | 30 | Sleep | Sedentary | between |  |
| -0.65 [-1.57, 0.27] | 30 | Standing | Sedentary | between |  |
| -0.95 [-3.76, 1.92] | 30 | LPA | Sedentary | between |  |
| 1.34 [-0.29, 2.94] | 30 | MVPA | Sedentary | between |  |
| 0.61 [-0.28, 1.46] | 30 | Sleep | Standing | between |  |
| 0.55 [-0.23, 1.33] | 30 | Sedentary | Standing | between |  |
| -0.41 [-3.57, 2.82] | 30 | LPA | Standing | between |  |
| 1.88 [-0.01, 3.77] | 30 | MVPA | Standing | between |  |
| 0.67 [-1.03, 2.3] | 30 | Sleep | LPA | between |  |
| 0.61 [-1.27, 2.42] | 30 | Sedentary | LPA | between |  |
| -0.05 [-2.37, 2.28] | 30 | Standing | LPA | between |  |
| 1.94 [-0.65, 4.43] | 30 | MVPA | LPA | between |  |
| -0.82 [-2.03, 0.37] | 30 | Sleep | MVPA | between |  |
| -0.89 [-2.01, 0.23] | 30 | Sedentary | MVPA | between |  |
| -1.54 [-3.08, -0.03] | 30 | Standing | MVPA | between | Yes |
| -1.84 [-4.92, 1.31] | 30 | LPA | MVPA | between |  |
| 0.11 [-0.01, 0.24] | 30 | Sedentary | Sleep | within |  |
| 0.11 [-0.19, 0.42] | 30 | Standing | Sleep | within |  |
| 0.49 [-0.43, 1.43] | 30 | LPA | Sleep | within |  |
| -0.1 [-0.49, 0.28] | 30 | MVPA | Sleep | within |  |
| -0.12 [-0.25, 0.01] | 30 | Sleep | Sedentary | within |  |
| 0 [-0.29, 0.31] | 30 | Standing | Sedentary | within |  |
| 0.38 [-0.54, 1.3] | 30 | LPA | Sedentary | within |  |
| -0.22 [-0.6, 0.16] | 30 | MVPA | Sedentary | within |  |
| -0.11 [-0.37, 0.14] | 30 | Sleep | Standing | within |  |
| 0.01 [-0.25, 0.25] | 30 | Sedentary | Standing | within |  |
| 0.39 [-0.72, 1.47] | 30 | LPA | Standing | within |  |
| -0.21 [-0.67, 0.24] | 30 | MVPA | Standing | within |  |
| -0.34 [-0.94, 0.25] | 30 | Sleep | LPA | within |  |
| -0.23 [-0.81, 0.35] | 30 | Sedentary | LPA | within |  |
| -0.23 [-1.02, 0.58] | 30 | Standing | LPA | within |  |
| -0.44 [-1.17, 0.29] | 30 | MVPA | LPA | within |  |
| 0.04 [-0.22, 0.31] | 30 | Sleep | MVPA | within |  |
| 0.16 [-0.1, 0.42] | 30 | Sedentary | MVPA | within |  |
| 0.15 [-0.24, 0.56] | 30 | Standing | MVPA | within |  |
| 0.54 [-0.46, 1.54] | 30 | LPA | MVPA | within |  |

## 1.3 5-minute reallocations

- Reallocation of 5 minutes between movement behaviours and
  *valence*

| Estimate [95% CI] | Minute | From | To | Level | Sig |
| --- | --- | --- | --- | --- | --- |
| -0.05 [-0.22, 0.12] | 5 | Sedentary | Sleep | between |  |
| -0.25 [-0.51, 0] | 5 | Standing | Sleep | between |  |
| -0.43 [-0.95, 0.07] | 5 | LPA | Sleep | between |  |
| 0.07 [-0.29, 0.42] | 5 | MVPA | Sleep | between |  |
| 0.05 [-0.12, 0.22] | 5 | Sleep | Sedentary | between |  |
| -0.2 [-0.42, 0.03] | 5 | Standing | Sedentary | between |  |
| -0.37 [-0.93, 0.19] | 5 | LPA | Sedentary | between |  |
| 0.12 [-0.23, 0.47] | 5 | MVPA | Sedentary | between |  |
| 0.25 [0, 0.5] | 5 | Sleep | Standing | between |  |
| 0.19 [-0.02, 0.41] | 5 | Sedentary | Standing | between |  |
| -0.18 [-0.84, 0.46] | 5 | LPA | Standing | between |  |
| 0.32 [-0.14, 0.74] | 5 | MVPA | Standing | between |  |
| 0.4 [-0.06, 0.88] | 5 | Sleep | LPA | between |  |
| 0.35 [-0.18, 0.87] | 5 | Sedentary | LPA | between |  |
| 0.15 [-0.46, 0.78] | 5 | Standing | LPA | between |  |
| 0.47 [-0.13, 1.11] | 5 | MVPA | LPA | between |  |
| -0.06 [-0.4, 0.28] | 5 | Sleep | MVPA | between |  |
| -0.12 [-0.44, 0.22] | 5 | Sedentary | MVPA | between |  |
| -0.31 [-0.72, 0.13] | 5 | Standing | MVPA | between |  |
| -0.49 [-1.15, 0.14] | 5 | LPA | MVPA | between |  |
| -0.03 [-0.07, 0.01] | 5 | Sedentary | Sleep | within |  |
| -0.07 [-0.14, 0] | 5 | Standing | Sleep | within |  |
| -0.04 [-0.2, 0.11] | 5 | LPA | Sleep | within |  |
| -0.18 [-0.27, -0.08] | 5 | MVPA | Sleep | within | Yes |
| 0.03 [-0.01, 0.07] | 5 | Sleep | Sedentary | within |  |
| -0.04 [-0.1, 0.01] | 5 | Standing | Sedentary | within |  |
| -0.01 [-0.17, 0.13] | 5 | LPA | Sedentary | within |  |
| -0.15 [-0.24, -0.06] | 5 | MVPA | Sedentary | within | Yes |
| 0.07 [0, 0.14] | 5 | Sleep | Standing | within |  |
| 0.04 [-0.01, 0.1] | 5 | Sedentary | Standing | within |  |
| 0.03 [-0.17, 0.21] | 5 | LPA | Standing | within |  |
| -0.1 [-0.22, 0] | 5 | MVPA | Standing | within |  |
| 0.04 [-0.1, 0.19] | 5 | Sleep | LPA | within |  |
| 0.01 [-0.12, 0.16] | 5 | Sedentary | LPA | within |  |
| -0.03 [-0.21, 0.16] | 5 | Standing | LPA | within |  |
| -0.13 [-0.31, 0.05] | 5 | MVPA | LPA | within |  |
| 0.17 [0.08, 0.26] | 5 | Sleep | MVPA | within | Yes |
| 0.14 [0.06, 0.22] | 5 | Sedentary | MVPA | within | Yes |
| 0.09 [-0.01, 0.2] | 5 | Standing | MVPA | within |  |
| 0.12 [-0.06, 0.31] | 5 | LPA | MVPA | within |  |

- Reallocation of 5 minutes between movement behaviours and
  *energetic arousal*

| Estimate [95% CI] | Minute | From | To | Level | Sig |
| --- | --- | --- | --- | --- | --- |
| 0.03 [-0.13, 0.19] | 5 | Sedentary | Sleep | between |  |
| -0.05 [-0.29, 0.2] | 5 | Standing | Sleep | between |  |
| -0.26 [-0.74, 0.22] | 5 | LPA | Sleep | between |  |
| 0.27 [-0.07, 0.64] | 5 | MVPA | Sleep | between |  |
| -0.03 [-0.19, 0.13] | 5 | Sleep | Sedentary | between |  |
| -0.08 [-0.29, 0.14] | 5 | Standing | Sedentary | between |  |
| -0.29 [-0.82, 0.24] | 5 | LPA | Sedentary | between |  |
| 0.25 [-0.09, 0.59] | 5 | MVPA | Sedentary | between |  |
| 0.05 [-0.19, 0.29] | 5 | Sleep | Standing | between |  |
| 0.08 [-0.13, 0.28] | 5 | Sedentary | Standing | between |  |
| -0.21 [-0.84, 0.41] | 5 | LPA | Standing | between |  |
| 0.32 [-0.09, 0.75] | 5 | MVPA | Standing | between |  |
| 0.24 [-0.21, 0.69] | 5 | Sleep | LPA | between |  |
| 0.27 [-0.23, 0.77] | 5 | Sedentary | LPA | between |  |
| 0.19 [-0.4, 0.79] | 5 | Standing | LPA | between |  |
| 0.51 [-0.08, 1.16] | 5 | MVPA | LPA | between |  |
| -0.26 [-0.6, 0.07] | 5 | Sleep | MVPA | between |  |
| -0.23 [-0.55, 0.08] | 5 | Sedentary | MVPA | between |  |
| -0.31 [-0.72, 0.09] | 5 | Standing | MVPA | between |  |
| -0.52 [-1.18, 0.1] | 5 | LPA | MVPA | between |  |
| -0.01 [-0.05, 0.03] | 5 | Sedentary | Sleep | within |  |
| 0.01 [-0.05, 0.08] | 5 | Standing | Sleep | within |  |
| -0.03 [-0.2, 0.14] | 5 | LPA | Sleep | within |  |
| -0.21 [-0.31, -0.11] | 5 | MVPA | Sleep | within | Yes |
| 0.01 [-0.03, 0.05] | 5 | Sleep | Sedentary | within |  |
| 0.02 [-0.04, 0.08] | 5 | Standing | Sedentary | within |  |
| -0.02 [-0.19, 0.16] | 5 | LPA | Sedentary | within |  |
| -0.2 [-0.3, -0.11] | 5 | MVPA | Sedentary | within | Yes |
| -0.01 [-0.08, 0.05] | 5 | Sleep | Standing | within |  |
| -0.02 [-0.08, 0.03] | 5 | Sedentary | Standing | within |  |
| -0.04 [-0.25, 0.16] | 5 | LPA | Standing | within |  |
| -0.23 [-0.34, -0.11] | 5 | MVPA | Standing | within | Yes |
| 0.03 [-0.13, 0.19] | 5 | Sleep | LPA | within |  |
| 0.02 [-0.14, 0.18] | 5 | Sedentary | LPA | within |  |
| 0.04 [-0.15, 0.24] | 5 | Standing | LPA | within |  |
| -0.19 [-0.39, 0.01] | 5 | MVPA | LPA | within |  |
| 0.2 [0.11, 0.3] | 5 | Sleep | MVPA | within | Yes |
| 0.19 [0.1, 0.28] | 5 | Sedentary | MVPA | within | Yes |
| 0.22 [0.11, 0.33] | 5 | Standing | MVPA | within | Yes |
| 0.17 [-0.03, 0.38] | 5 | LPA | MVPA | within |  |

- Reallocation of 5 minutes between movement behaviours and
  *calmness*

| Estimate [95% CI] | Minute | From | To | Level | Sig |
| --- | --- | --- | --- | --- | --- |
| -0.09 [-0.26, 0.07] | 5 | Sedentary | Sleep | between |  |
| -0.37 [-0.63, -0.12] | 5 | Standing | Sleep | between | Yes |
| -0.35 [-0.87, 0.16] | 5 | LPA | Sleep | between |  |
| 0.15 [-0.21, 0.49] | 5 | MVPA | Sleep | between |  |
| 0.1 [-0.07, 0.26] | 5 | Sleep | Sedentary | between |  |
| -0.28 [-0.5, -0.06] | 5 | Standing | Sedentary | between | Yes |
| -0.25 [-0.84, 0.3] | 5 | LPA | Sedentary | between |  |
| 0.24 [-0.11, 0.57] | 5 | MVPA | Sedentary | between |  |
| 0.37 [0.12, 0.61] | 5 | Sleep | Standing | between | Yes |
| 0.27 [0.06, 0.48] | 5 | Sedentary | Standing | between | Yes |
| 0.02 [-0.65, 0.66] | 5 | LPA | Standing | between |  |
| 0.51 [0.11, 0.93] | 5 | MVPA | Standing | between | Yes |
| 0.33 [-0.14, 0.82] | 5 | Sleep | LPA | between |  |
| 0.24 [-0.28, 0.79] | 5 | Sedentary | LPA | between |  |
| -0.04 [-0.65, 0.59] | 5 | Standing | LPA | between |  |
| 0.48 [-0.15, 1.1] | 5 | MVPA | LPA | between |  |
| -0.13 [-0.46, 0.2] | 5 | Sleep | MVPA | between |  |
| -0.23 [-0.54, 0.11] | 5 | Sedentary | MVPA | between |  |
| -0.51 [-0.91, -0.12] | 5 | Standing | MVPA | between | Yes |
| -0.48 [-1.13, 0.17] | 5 | LPA | MVPA | between |  |
| 0 [-0.03, 0.03] | 5 | Sedentary | Sleep | within |  |
| -0.01 [-0.08, 0.05] | 5 | Standing | Sleep | within |  |
| -0.19 [-0.36, -0.02] | 5 | LPA | Sleep | within | Yes |
| 0.02 [-0.07, 0.11] | 5 | MVPA | Sleep | within |  |
| 0 [-0.03, 0.03] | 5 | Sleep | Sedentary | within |  |
| -0.01 [-0.07, 0.05] | 5 | Standing | Sedentary | within |  |
| -0.19 [-0.35, -0.03] | 5 | LPA | Sedentary | within | Yes |
| 0.02 [-0.07, 0.11] | 5 | MVPA | Sedentary | within |  |
| 0.01 [-0.05, 0.08] | 5 | Sleep | Standing | within |  |
| 0.01 [-0.05, 0.07] | 5 | Sedentary | Standing | within |  |
| -0.18 [-0.38, 0.02] | 5 | LPA | Standing | within |  |
| 0.03 [-0.08, 0.14] | 5 | MVPA | Standing | within |  |
| 0.18 [0.02, 0.33] | 5 | Sleep | LPA | within | Yes |
| 0.18 [0.02, 0.33] | 5 | Sedentary | LPA | within | Yes |
| 0.17 [-0.02, 0.36] | 5 | Standing | LPA | within |  |
| 0.2 [0.01, 0.39] | 5 | MVPA | LPA | within | Yes |
| -0.02 [-0.1, 0.07] | 5 | Sleep | MVPA | within |  |
| -0.02 [-0.1, 0.07] | 5 | Sedentary | MVPA | within |  |
| -0.03 [-0.13, 0.08] | 5 | Standing | MVPA | within |  |
| -0.21 [-0.41, -0.01] | 5 | LPA | MVPA | within | Yes |

- Reallocation of 5 minutes between movement behaviours and
  *working memory performance*

| Estimate [95% CI] | Minute | From | To | Level | Sig |
| --- | --- | --- | --- | --- | --- |
| -0.01 [-0.12, 0.1] | 5 | Sedentary | Sleep | between |  |
| -0.11 [-0.26, 0.05] | 5 | Standing | Sleep | between |  |
| -0.14 [-0.48, 0.22] | 5 | LPA | Sleep | between |  |
| 0.17 [-0.07, 0.41] | 5 | MVPA | Sleep | between |  |
| 0.01 [-0.1, 0.12] | 5 | Sleep | Sedentary | between |  |
| -0.1 [-0.24, 0.04] | 5 | Standing | Sedentary | between |  |
| -0.13 [-0.5, 0.26] | 5 | LPA | Sedentary | between |  |
| 0.18 [-0.04, 0.4] | 5 | MVPA | Sedentary | between |  |
| 0.11 [-0.05, 0.26] | 5 | Sleep | Standing | between |  |
| 0.1 [-0.04, 0.24] | 5 | Sedentary | Standing | between |  |
| -0.03 [-0.47, 0.42] | 5 | LPA | Standing | between |  |
| 0.28 [0, 0.56] | 5 | MVPA | Standing | between |  |
| 0.13 [-0.2, 0.44] | 5 | Sleep | LPA | between |  |
| 0.12 [-0.24, 0.47] | 5 | Sedentary | LPA | between |  |
| 0.02 [-0.41, 0.44] | 5 | Standing | LPA | between |  |
| 0.3 [-0.14, 0.72] | 5 | MVPA | LPA | between |  |
| -0.16 [-0.38, 0.07] | 5 | Sleep | MVPA | between |  |
| -0.17 [-0.38, 0.04] | 5 | Sedentary | MVPA | between |  |
| -0.27 [-0.54, 0] | 5 | Standing | MVPA | between |  |
| -0.3 [-0.73, 0.16] | 5 | LPA | MVPA | between |  |
| 0.02 [0, 0.04] | 5 | Sedentary | Sleep | within |  |
| 0.02 [-0.03, 0.07] | 5 | Standing | Sleep | within |  |
| 0.07 [-0.06, 0.19] | 5 | LPA | Sleep | within |  |
| -0.01 [-0.07, 0.04] | 5 | MVPA | Sleep | within |  |
| -0.02 [-0.04, 0] | 5 | Sleep | Sedentary | within |  |
| 0 [-0.05, 0.05] | 5 | Standing | Sedentary | within |  |
| 0.05 [-0.07, 0.17] | 5 | LPA | Sedentary | within |  |
| -0.03 [-0.08, 0.02] | 5 | MVPA | Sedentary | within |  |
| -0.02 [-0.06, 0.03] | 5 | Sleep | Standing | within |  |
| 0 [-0.04, 0.04] | 5 | Sedentary | Standing | within |  |
| 0.05 [-0.1, 0.2] | 5 | LPA | Standing | within |  |
| -0.03 [-0.1, 0.04] | 5 | MVPA | Standing | within |  |
| -0.06 [-0.18, 0.05] | 5 | Sleep | LPA | within |  |
| -0.04 [-0.16, 0.07] | 5 | Sedentary | LPA | within |  |
| -0.05 [-0.19, 0.1] | 5 | Standing | LPA | within |  |
| -0.08 [-0.21, 0.06] | 5 | MVPA | LPA | within |  |
| 0.01 [-0.04, 0.06] | 5 | Sleep | MVPA | within |  |
| 0.03 [-0.02, 0.08] | 5 | Sedentary | MVPA | within |  |
| 0.03 [-0.04, 0.1] | 5 | Standing | MVPA | within |  |
| 0.08 [-0.06, 0.22] | 5 | LPA | MVPA | within |  |

## 1.4 Visualisation of reallocations from 1 to 60 minutes

*Notes.* Significance at a: 5 minutes, b: 30 minutes, and c:
60 minutes.

### 1.4.1 Between person level

### 1.4.2 Within person level
